# Probabilistic and machine learning-based retrieval approaches for biomedical dataset retrieval

**DOI:** 10.1093/database/bax104

**Published:** 2018-03-28

**Authors:** Payam Karisani, Zhaohui S Qin, Eugene Agichtein

**Affiliations:** 1 Department of Computer Science, Mathematics & Science Center, Emory University, Suite W401, 400 Dowman Drive NE, Atlanta, Georgia 30322, USA; 2 Department of Biostatistics and Bioinformatics, Emory University, 1518 Clifton Road NE, Atlanta, Georgia 30322-4201, USA

## Abstract

The bioCADDIE dataset retrieval challenge brought together different approaches to retrieval of biomedical datasets relevant to a user’s query, expressed as a text description of a needed dataset. We describe experiments in applying a data-driven, machine learning-based approach to biomedical dataset retrieval as part of this challenge. We report on a series of experiments carried out to evaluate the performance of both probabilistic and machine learning-driven techniques from information retrieval, as applied to this challenge. Our experiments with probabilistic information retrieval methods, such as query term weight optimization, automatic query expansion and simulated user relevance feedback, demonstrate that automatically boosting the weights of important keywords in a verbose query is more effective than other methods. We also show that although there is a rich space of potential representations and features available in this domain, machine learning-based re-ranking models are not able to improve on probabilistic information retrieval techniques with the currently available training data. The models and algorithms presented in this paper can serve as a viable implementation of a search engine to provide access to biomedical datasets. The retrieval performance is expected to be further improved by using additional training data that is created by expert annotation, or gathered through usage logs, clicks and other processes during natural operation of the system.

**Database URL**: https://github.com/emory-irlab/biocaddie

## Background and motivation 

With rapid technological development such as DNA sequencing and brain imaging, ever increasing volumes of massive datasets have been produced. Data sharing policies mandated by agencies such as the NIH, have encouraged high throughput experimental data to accumulate rapidly in public repositories. As an example, the NCBI Gene Expression Omnibus has to-date (November 2017) archived >91 000 experimental studies, which comprise >2 million samples.

Such massive amounts of openly accessible data offer unprecedented opportunities to advance our understanding of biology, human health and diseases. There is little doubt that biomedicine, like many other disciplines, are accelerating into a new era of Big Data. However, along with exciting prospects there are enormous challenges. It is impossible to conveniently browse through datasets collected from millions of experiments. In a perspective article, which describes NIH’s vision of Big Data to Knowledge (BD2K) (1), Margolis *et al.* pointed out that ‘A fundamental question for BD2K is how to enable the identification, access and citation of (i.e. credit for) biomedical data.’ In Eric Green’s presentation on ‘NIH and Biomedical ‘Big Data,’ the first ‘major problems to solve’ for big data is ‘Locating the data.’ This is the challenge on which we focus in this paper: developing and evaluating techniques for finding *relevant* biomedical datasets.

Fortunately, many techniques for searching online sources have been developed for the Web, which could be adapted to search for biomedical datasets.

While biomedical literature remains the dominating source of biomedical knowledge, the explosion of massive biomedical data offers an attractive, alternative source for biomedical knowledge since these assays provide somewhat unbiased (no vetting from investigators), comprehensive view of the study subject. However, to maximally exploit the new information source, key informatics infrastructure needs to be developed. One of the major aims of the recent BD2K (https://datascience.nih.gov/bd2k) efforts (e.g. bioCADDIE) is focused on making biomedical data searchable and reusable to speed up discovery (2). To compare different approaches to retrieving biomedical datasets in an objective and uniform fashion, bioCADDIE investigators organized the ‘BioCADDIE Retrieval Challenge,’ which provided a small amount of ‘training’ data (user queries and lists of dataset results, marked relevant or non-relevant for the query), as well as a static ‘corpus’ – i.e. a snapshot of the datasets in 20 different online repositories. The participating systems were evaluated on accuracy of retrieving datasets for hidden ‘test’ queries. More details about the Challenge are provided in the bioCADDIE Challenge description articles, currently under review for the Database journal (3, 4).

In this article, we describe our approach, system and experiments in addressing the bioCADDIE Retrieval Challenge task. Our system draws on a variety of information retrieval techniques, and comprises of three main steps in the retrieval process. First, the original user query, expressed as a textual description, is used to retrieve the initial set of datasets. For this step, the system matches the query to the dataset description, extracted from the website from which the dataset was drawn. Specifically, the query words are matched against the available text fields (i.e. the dataset title, text and description). Second, by analysing the top retrieved datasets, and the relevant *external* resources available online, the system automatically *reformulates* and *expands* the original query to build a more informative query. The expanded query is used to retrieve a second list of datasets. Finally, the system extracts an additional, more comprehensive list of *features* to describe each dataset and its match to the query (e.g. textual similarity of the query to each of the text fields). This expanded representation of the query and dataset is then used in a machine-learned ranking, by using a learning to rank framework to generate the final ranked list of datasets.

One technique from information retrieval which has been effective in improving a match of a query to relevant datasets is called ‘Blind Relevance Feedback’ (BRF) (11), which analyses the top retrieved documents to identify frequent shared terms, which in turn could be used to expand the query to retrieve more documents like it. The BRF technique is described in detail in the Query reformulation and expansion section. Our experiments show that if the user query is long, automatic BRF is less effective than detecting important query terms and boosting (automatically increasing) their weights. Another technique is to automatically learn the importance of query terms, and other characteristics of documents and their matches to a query, by using a machine-learned ranking (LTR) (5, 6). LTR models automatically learn to weight query-dataset match features for an improved ranking function. However, we find that with the available (small) amounts of expert-annotated training data, LTR model does not improve the relevance of the results, compared to the optimized probabilistic information retrieval methods. However, our experiments also indicate that by using additional, potentially erroneous (noisy) judgements for training data, the quality of the LTR model (described in the Learning to rank section) learned over the training set can be significantly improved and the LTR model becomes more robust for new, unseen queries. This is an important finding, since implicit feedback, such as user clicks on the results in the retrieved list, can be considered a type of noisy judgement and can be used to further improve the effectiveness of the LTR framework continuously, after the system is deployed.

## bioCADDIE challenge overview

The bioCADDIE dataset retrieval challenge (3) aims to develop an infrastructure for building a unified system that can facilitate the access to the variety of datasets built by medical researchers (primarily, bioinformatics researchers.) The participants were provided with a training corpus containing 794 992 dataset descriptions, crawled from 20 different web domains (4). Each dataset initially contained the following fields:

- DOCNO: unique dataset ID, which is used to evaluate the relevance of the result.
- TITLE: dataset title, part of the textual description.
- REPOSITORY: source of the dataset.
- TEXT (In the original corpus this field is named METADATA, but for clarity we name this field TEXT to distinguish from other fields.): textual detailed description of the dataset.

As we will describe in the Indexing and initial retrieval section, we further expanded and enriched the dataset description with external resources.

**Training data:** In order to optimize the retrieval systems prior to the official Challenge date, a small amount of training data was provided, with expert labels of judgements describing the relevance of top datasets, retrieved by a commercial search system, ElasticSearch, for each training query. More details on the dataset construction are described in reference (3). Due to the high cost of manually labeling the relevance of the datasets to the queries by biomedical experts, only six queries with relevance judgments were provided for training of the systems, which is lower than previous information retrieval challenges. Additional 30 queries, without relevance judgments, were also provided for training, to give a better idea of the *kind* of queries users might ask.

**Test data:** In the test phase of the Challenge, 15 queries, without any relevance judgments were provided. The systems then retrieved up to 100 ranked results for each of the test queries, and submitted these results to the Challenge organizers for expert evaluation. The retrieved datasets were rated by the expert judges in the scale of 0 to 2, where 0 (‘Poor’) indicates an irrelevant dataset and 2 (‘Excellent’) indicates a very relevant dataset to the query.

**Evaluation Metrics:** to evaluate the judged results, the bioCADDIE Challenge organizers used established metrics from information retrieval literature, namely infNDCG, MAP and Precision at 1 and at 10. The infNDCG metric from information retrieval (7) estimates the value of the more commonly used NDCG metric, which gives increasing discounts for relevance of results further down the ranked list, through stratified random sampling and is expected to be more robust in the presence of incomplete judgements (NDCG, MAP and P@10 are described in detail in the Experimental setup section). More details on the test and judging process is described in reference (4).

### Challenges of the bioCADDIE retrieval challenge for information retrieval

The bioCADDIE challenge differs from traditional information retrieval tasks in multiple ways. First, the query intent is to find a useful dataset to potentially use for research, not to learn about the research topic, as in more traditional information retrieval. Second, the corpus and document characteristics are different from traditional text or Web documents, which made for higher rate of query-document term mismatch. Finally, the amount of available training data is lower than in other informational retrieval tasks. We describe these challenges in detail below.

**Query intent and corpus characteristics:** Unlike traditional information retrieval tasks, where the majority of the queries are informational (i.e. information need can be satisfied by reading documents), the queries in bioCADDIE challenge are primarily ‘transactional,’ (i.e. the query intent is to find useful data to do some processing to answer a question and the answer not contained in the document explicitly). Additionally, due to the nature of the datasets, and high degree of specialization and domain knowledge in biomedical research, the dataset descriptions often do not explicitly mention the related research areas.

**Query and document mismatch:** The key terms in the query often do not appear in the relevant dataset descriptions, suggesting the necessity for query reformulation and expansion.

**Training data:** A very small number of training queries presents difficulties during the development and system tuning phases. This presents larger difficulties for the machine learning-based techniques (LTR) (5) which are heavily dependent on the availability of rich and extensive training data. However, we emphasize the effort that the bioCADDIE Challenge organizers expanded in preparing this dataset. Obtaining expert judgments from biomedical researchers is an expensive and time-consuming process, and thus the amount of the training and test data provided is representative of what can be expected from replicating the Retrieval Challenge in other biomedical retrieval domains.

## Methodology: retrieval system architecture and implementation

**Figure 1.**
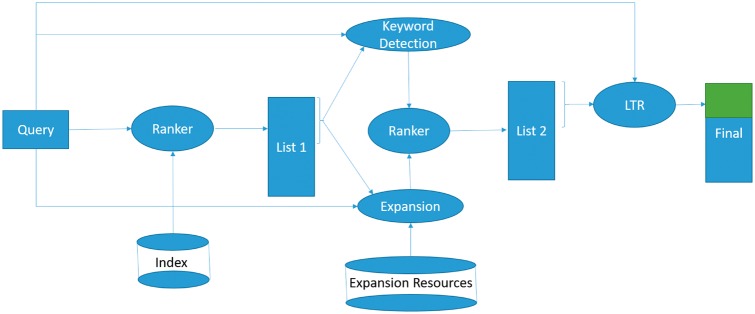
The system architecture and data flow diagram for biomedical dataset retrieval and bioCADDIE challenge.

In this section, we describe the system architecture and key design decisions. The system architecture and data flow diagram is depicted in Figure 1. The diagram illustrates that the provided query is used to retrieve the initial set of datasets, and to access the relevant external resources (described in the Indexing and initial retrieval section). The discovered terms and a keyword detection algorithm are leveraged to reformulate the query, and a new set of datasets is retrieved using the new query (discussed in the Query reformulation and expansion section). The resulting list is used to extract additional features, used in a learning to rank model to derive the final dataset ranking (the Learning to rank section).

Below we discuss the main design decisions that were made in the development phase, and in each case, we also discuss how it may potentially address the problems mentioned in the section bioCADDIE challenge overview.

### Indexing and initial retrieval

Each dataset in the bioCADDIE corpus consists of two main text sections, TITLE and TEXT. Our preliminary experiments showed that the sections are complementary, therefore, we decided to index each section separately and train the retrieval model to learn the best settings. Additionally, as we discussed in the bioCADDIE challenge overview section, due to the high degree of specialization and domain knowledge in building each dataset, these fields often do not explicitly mention the related research areas. Consequently, this may cause a high degree of query document mismatch. To address this issue, we augmented the datasets with ‘meta-data,’ retrieved from the description of the source database description online (e.g. the ‘About’ page from where the datasets were drawn), which may contain the missing information to describe which questions the datasets *could* answer [Although due to the small number of repositories (20 sources) we manually collected the mentioned webpages, with minimal effort this process could be automated to collect the descriptions automatically.] This additional information was added to the METADATA field of the datasets, as the third text field. This data was extracted once for each of the online repositories, and automatically added to each of the dataset descriptions (as a METADATA field) during the system indexing of the datasets. No manual curation or filtering of this field was performed. Finally, the retrieval model was trained to learn the best parameter settings for the initial retrieval step (‘List 1’ in Figure 1). We used Apache Lucene as the indexing and retrieval engine. To incorporate dataset fields, we used Lucene multiple field search. More details about the implementation decisions can be found in the Experimental setup section.

### Query reformulation and expansion

We approached query reformulation in two ways: Query term weighting, and expansion with new terms.

**Query term weighting.** The queries prepared by the bioCADDIE organizers are relatively *verbose*, averaging 15.8 terms (words) per query. Query term weighting and keyword detection for such verbose queries have shown to be effective in previous work (8, 9). We used one such technique, called Weighted Information Gain (*WIG*), introduced by Zhou and Croft in reference (10), to detect the most important keywords in the query and boost (assign a higher weight to) these terms. *WIG* captures the change in information when an average document is retrieved to the state when the actual documents are retrieved. Thus, we expect the *WIG* model to assign a higher weight to the most important keywords in the query. More formally, *WIG* is defined as follows:

(1) $$wig(q_{i})=\frac{\frac{1}{N}\sum_{d\in T_{N}(q_{i})}\;\text{log}\;p(q_{i}|d)-\text{log}\;p(q_{i}|C)}{-\text{log}\;p(q_{i}|C)}$$

where *q_i_* denotes the *i-th* query term, *wig(q_i_)* the assigned weight to *q_i_*, *T_N_(q_i_)* the top documents retrieved in response to *q_i_*, *N* the number of the selected top documents, *p(q_i_ |d)* the smoothed maximum likelihood of observing *q_i_* in document *d* and finally *p(q_i_ |C)* the probability of observing *q_i_* in the whole corpus *C*, and can be computed as follows:

(2) $$p(q_{i}|C)=\frac{F_{i}}{\sum_{j}F_{j}}$$

where *F_i_* is the total frequency of observing *q_i_* in the whole corpus. For query term weighting, we used the default approach of Lucene (called boosting), which multiplies the final contribution (to the score of a document) of each query term by the weight which is assigned to that query term.

**Automated Query Expansion with new terms.** As discussed, one of the key challenges for dataset retrieval is the mismatch between the terms in the user query, and the terms in the dataset title or description. A well-known technique in information retrieval is to perform *automatic query expansion*, where new terms are added to the query that is expected to match additional relevant datasets. For this, we used *internal* and *external* resources for query expansion.

As an *internal* query expansion method, we used a method called BRF (11), which assumes that the top retrieved documents are relevant and adds the most frequent words in these documents to the query.

As an *external* query expansion method, we used two biomedical databases, specifically Kyoto Encyclopedia of Genes and Genomes (KEGG) (Available at: http://www.genome.jp/kegg/kegg1.html) and HGNC (Available at: http://www.genenames.org/), as well as restricted Web search and PubMed search. KEGG is a large online database resource that integrates disease and genomic information, especially with the link to pathway and individual gene information. KEGG API allows detecting whether a given word is a gene name, a pathway name, or disease name. Our approach categorizes each word in the query, and then retrieves the most relevant information related to each of the keywords recognized by the API, in order to expand the original query with extended retrieved contexts. For example, for a query containing disease keywords, we only expand the query with highly relevant information with this disease.

Additionally, a commercial vertical search engine (A search engine which is restricted to retrieve documents from a specific web domain.) is used to retrieve the top-ranked pages of Wikipedia and NCBI websites. To avoid query drift (i.e. changing the meaning of the query by introducing non-relevant terms into the expanded query), we restricted the candidate terms to those which appear at least once in the initial top datasets. Furthermore, we used a list of manually collected medical terms (This list is different from the stopwords list, it consists of 14 000 medical terms and is used to prevent the expansion of non-medical terms.) to restrict querying the KEGG, HGNC and the search engine. The final query expansion terms are selected based on the frequency of their occurrences in the ‘pseudo-document,’ created by concatenating all the retrieved external resources into a single text ‘document.’ The new, expanded query is issued against the index to retrieve the second rank list (‘List 2’ in Figure 1).

### Learning to rank

Augmenting the original user query with new terms usually improves *recall* (i.e. the fraction of relevant documents in the returned list), because new terms help the retrieval model to match query with documents which may not contain any of the original query terms. However, this may reduce *precision* (i.e. the fraction of relevant documents contained in the returned list), since the documents matching the augmented query may not be relevant to the original query. Furthermore, the new expanded query terms may be spurious, because the automated query expansion algorithms are never perfect.

Using a machine-learned ranking, specifically the learning to rank (LTR) framework, is a way to address this issue, by introducing a richer, more comprehensive representation of the query match with the dataset, which goes beyond individual keyword overlap. Many algorithms, that aim to learn the importance of the features, have been proposed, and we describe them in detail below. The majority of them use the same feature representation, summarized in Table 1. The features are categorized into eight groups based on their shared attributes. In the description column, *t_i_* indicates the *i-th* common term in query *q* and dataset *d*.

**Table 1.** Features used to describe query-dataset match for the Learning to Rank (LTR) machine learned ranking

Group No	Feature name	Description
1	*BM25*	BM25 similarity score of the whole dataset
1	*BM25Title*	BM25 similarity score of the TITLE
1	*BM25Text*	BM25 similarity score of the TEXT
1	*BM25Meta*	BM25 similarity score of the METADATA
2	*1GramTFTitle*	$\sum_{t_{i}\in q\cap d}TF(t_{i},d)$ in TITLE (sum of frequencies of matching terms in dataset title field)
2	*1GramTFText*	$\sum_{t_{i}\in q\cap d}TF(t_{i},d)$ in TEXT (““““““in text field)
2	*1GramTFMeta*	$\sum_{t_{i}\in q\cap d}TF(t_{i},d)$ in METADATA (““““““in metadata field)
3	*1GramIDFTitle*	$\sum_{t_{i}\in q\cap d}IDF(t_{i},d)$ in TITLE (sum of inverse frequencies of matching terms in dataset title field)
3	*1GramIDFText*	$\sum_{t_{i}\in q\cap d}IDF(t_{i},d)$ in TEXT (“”“”“”in dataset text field)
3	*1GramIDFMeta*	$\sum_{t_{i}\in q\cap d}IDF(t_{i},d)$ in METADATA (“”“”“”in dataset metadata field)
4	*1GramTFIDFTitle*	$\sum_{t_{i}\in q\cap d}TF(t_{i},d).IDF(t_{i},d)$ in TITLE (sum of TF*IDF scores of matching terms in dataset title field)
4	*1GramTFIDFText*	$\sum_{t_{i}\in q\cap d}TF(t_{i},d).IDF(t_{i},d)$ in TEXT (sum of TF*IDF scores of matching terms in dataset text field)
4	*1GramTFIDFMeta*	$\sum_{t_{i}\in q\cap d}TF(t_{i},d).IDF(t_{i},d)$ in METADATA (sum of TF*IDF scores of matching terms in dataset metadata field)
5	*1GramTFWhole*	$\sum_{t_{i}\in q\cap d}TF(t_{i},d)$ in the whole (concatenated) dataset
5	*1GramIDFWhole*	$\sum_{t_{i}\in q\cap d}IDF(t_{i},d)$ in the whole (concatenated) dataset
5	*1GramTFIDFWhole*	$\sum_{t_{i}\in q\cap d}TF(t_{i},d).IDF(t_{i},d)$ in the whole (concatenated) dataset
6	*2GramsTitle*	Number of common word 2-grams in the query and TITLE
6	*2GramsText*	Number of common word 2-grams in the query and TEXT
6	*2GramsMeta*	Number of common word 2-grams in the query and METADATA
6	*2GramsWhole*	Number of common word 2-grams in the query and the whole dataset
7	*DistanceFromStart*	Position of the first query term in the dataset TEXT field
8	*DomainWeight*	Ratio of the number of datasets belong to the dataset’s web domain to the whole datasets in the corpus

The commonly used features used in LTR framework are TF (Term Frequency), IDF (Inverse Document Frequency), BM25 score and their derivatives. The weight of the term in a document is captured through *TF* (term frequency), and is estimated by the frequency of the term. *IDF* (inverse document frequency) is used to capture the ‘importance’ of the term in the whole collection. There are multiple variants for IDF, we used the following:

(3) $$IDF_{i}=\text{log}(\frac{N}{n_{i}+1})+1$$

where *N* is the number of documents in the collection, and *n_i_* is the number of the documents that contain term *t_i_*. *IDF* score is higher for less frequent, and therefore more important terms in the collection. The statistical interpretation of the metric can be found in (12). *BM25* retrieval score (13) is an extension of the classic probabilistic model, and attempts to estimate the probability that the document and the query are relevant. To achieve higher accuracy, the *BM25* model *normalizes* the *TF* scores by using the document length, and uses additional tuning parameters *b* and *K1*. A thorough analysis of the model is reported in (13). In addition to the common features, we also used *DistanceFromStart*, to quantify the position of the first observed query term in the dataset TEXT to give a higher weight to the description introductions. *DomainWeight* feature computes the relative size of the source database from which the dataset was retrieved, as the fraction of number of datasets in the source database from all datasets, in order to boost the score of datasets drawn from larger, potentially more important, databases.

Although LTR has proven to be effective in other research areas (14), training set size has a large impact on the final results. We will investigate the cases where LTR can be potentially leveraged to improve result quality in the empirical portion of the paper.

**LTR Implementation**: We used the RankLib (Available at: https://sourceforge.net/p/lemur/wiki/RankLib/) library to implement the learning to rank framework. Several LTR algorithms were investigated: MART (15), RankNet (16) and Co-ordinate Ascent (17). MART (Multiple Additive Regression Trees) tries to learn a regression tree as ranking model. It combines boosting paradigm with regression trees so that it can have all the advantages of tree-based models, while overcoming the inaccuracy of these models. RankNet is a pairwise method, with Cross Entropy cost function to minimize the number of incorrectly ranked documents. It employs a neural network with Gradient Descent to learn the ranking, via minimizing the cost function. Co-ordinate Ascent is another, alternative method for learning a ranking. It is an iterative algorithm to optimize a multivariate function by doing one-dimensional search over a variable while fixing all the other variables. It is a list-wise method, and is known for good generalizations properties. We used all of these methods (with the recommended settings) to re-rank the top 100 datasets in the second list (in Figure 1), and to generate the final list of result datasets. In the Experiments section, we report the results achieved by the MART algorithm, since we did not observe a significant improvement in using other LTR methods.

## Experiments

We describe the training phase of the system in the Experimental setup section, and report the results in the Results section. We discuss and analyse the results, and report observations in the Discussion section.

### Experimental setup

We used the Apache Lucene system to pre-process, index and retrieve the dataset descriptions. Porter stemmer, and the Lucene default stop words were used to pre-process the dataset descriptions and queries. For each dataset, we stored the TITLE, TEXT and METADATA as described in the Methodology: retrieval system architecture and implementation section. BM25 model was used to retrieve the initial datasets. Lucene multiple field search was used to do query-term matching over all the three parts of the datasets.

For performance evaluation, we use *P@10*, *MAP* and *NDCG* metrics. *P@10* is precision (or the fraction of the relevant documents) retrieved in the top 10 documents. This metric measure the user’s satisfaction, since users often judge the results based solely on the top documents. *MAP* (Mean Average Precision) is an attempt to summarize the overall ranking in a single number. The main idea is to compute the average from all the precision values after observing each relevant document in the rank list. One limitation of MAP is that it is a binary based value, and cannot distinguish between the systems that retrieve the higher relevant documents first. *NDCG* (Normalized Discounted Cumulative Gain), a metric to incorporate graded relevancy, is used to compare different results for different queries. NDCG is extended from Cumulative Gain, which is the sum of the graded relevancies for the retrieved documents; and Discounted Cumulative Gain (DCG), which is the discounted values of the relevancy gains, so that those documents which are retrieved first are weighted more.

The dataset descriptions tended to be short, and contain specialized language, which required domain-specific parameter tuning. The official training, and test queries (the sum total of 21 queries) were used in a 4-fold cross validation such that the system was trained on 15–16 queries, and tested on the remaining ‘test fold’ queries, where in each test fold there were at least five queries. The reported results in the next sections are the averages over the folds and optimized for the highest NDCG. Grid search was used to optimize the parameters in each training fold. To reduce the grid search complexity, we optimized the initial retrieval—which includes the parameters in Table 2—over the official training queries, and fixed them for the subsequent cross validation experiments. Table 2 summarizes the tuned parameters, the corresponding investigated ranges and their optimal values.

**Table 2.** Initial (first phase) retrieval model parameters, with the range of values and the empirically tuned best value for each parameter

Parameter	Description	Range	Best value
*TITLE weight*	Weight of TITLE in the retrieval	0.1, 0.3, 0.5, 0.7	0.1
*TEXT weight*	Weight of TEXT in the retrieval	0.1, 0.3, 0.5, 0.7	0.3
*METDATA weight*	Weight of METADATA in the retrieval	0.1, 0.3, 0.5, 0.7	0.5
*BM25 k1*	K1 parameter in BM25	0.6, 1, 1.4, 1.8	1.8
*BM25 b*	B parameter in BM25	0.3, 0.5, 0.7, 0.9	0.7

Table 2 shows that, among dataset fields, the highest weight is assigned to the METADATA which signifies the importance of this field. BM25 parameters show that document length normalization is crucial in the retrieval step (with the value of b close to 1). Table 3 reports the tuned parameters, the corresponding investigated ranges and their optimal values for the query reformulation steps in the cross validation experiment. The optimal values were chosen based on their occurrence frequency in the folds.

**Table 3.** Query reformulation parameters, with the range of values and the empirically tuned best value for each parameter

Parameter	Description	Range	Best value
*Top datasets*	Top datasets selected for WIG model, BRF and external expansion	5, 10, 30	5
*Internal terms*	Number of terms added to the query by BRF	5, 10, 30	5
*Weights for internal terms*	Weight of the terms selected by BRF	0.1, 0.3, 0.5	0.1
*External terms*	Number of terms added to the query using external resources	5, 10, 30	10
*Weights for external terms*	Weight of the terms added using external resources	0.1, 0.3, 0.5	0.5

In order to reduce the complexity and search space for parameter optimization, we set the number of top datasets for *WIG* model, *BRF* and *external expansion* (all described in the Indexing and initial retrieval section) to the same value. We found that the optimal number of datasets for expansion is five, thus making our model somewhat conservative in including only a small number of pseudo-relevant datasets for query expansion. We also observe that the number of *external terms* and their corresponding weights is larger than their internal counterparts, signifying the importance of external resources in the expansion process.

To simplify training, we fixed all the parameters in Tables 2 and 3 before training the LTR framework.

### Results

**Figure 2.**
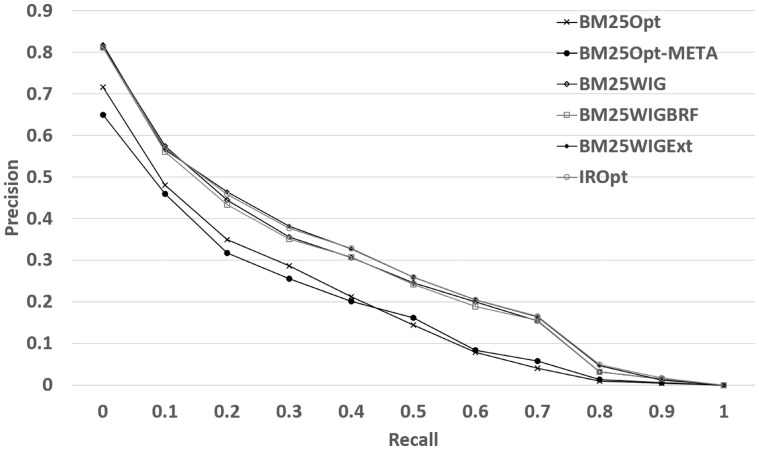
Retrieval performance of the query reformulation extensions.

Table 4 reports the performance results for the 4-fold cross validation experiment described in the previous section. Table 4 shows that the most effective measure to take is the query term weighting, which has the highest *NDCG* improvement (Improvement is computed based on the percentage change: 100 × (new*_*value – original_value) / original_value) (17.06%) over the original retrieval model. This improvement can be explained as follows: using query term weighting the retrieval model can detect the most informative words and discard the query terms which may potentially match irrelevant documents (see Discussion section for some examples). The results also indicate that given the verbose queries, and in the presence of an effective keyword detection method, we are unable to gain a significant benefit from the *BRF* expansion method. However, using external resources, conditional on the presence of the new terms in the top datasets, we observe an additional improvement (19.69% in NDCG) when using query expansion with external resources. Combining both expansion methods does not lead to a significantly better performance (we will refer to this run as *IROpt* in the following sections). The experiment also shows that if we remove METADATA field (row *BM25Opt-META* in Table 4), we observe a 2.4% degradation in *NDCG*. This signifies that the METADATA field on average helps to reduce the mismatch between the query and the dataset descriptions. Figure 2 demonstrates the precision-recall plots for the models reported in Table 4. We can observe that *WIG* extension (*BM25Wig*) is highly correlated with *BRF* extension (*BM25WigBRF*). The precision of both methods start to decline at recall 0.1, while using external resources slows the decline.

**Table 4.** Performance results for the steps described in the Methodology: retrieval system architecture and implementation section

Model	NDCG	MAP	P@10
*BM25Opt*: Optimized BM25	0.457	0.187	0.499
*BM25Opt-META*: Optimized BM25 – METADATA field	0.446	0.180	0.463
*BM25Wig:* Optimized BM25 + WIG model	0.535	0.261	0.601
*BM25WigBRF:* Optimized BM25 + WIG model + Expansion with BRF (1)	0.534	0.259	**0.606**
*BM25WigExt:* Optimized BM25 + WIG model + external terms (2)	0.547	**0.272**	0.590
*IROpt:* Optimized BM25 + WIG model + Expansion with (1) and (2)	**0.549**	**0.272**	0.586

The bold numbers indicate the highest achieved performance.

Table 5 shows the performance changes when we used LTR. The results show that applying LTR in the scarce training data environment causes overfitting, and the final model causes 5.1% degradation in NDCG, compared to the *IROpt* (The reason that we observe some difference in *IROpt* models in Tables 4 and 5 is that, as mentioned in the section Experimental setup, for the LTR part we fixed all the information retrieval parameters in Tables 2 and 3 and assumed there is a universal tuned parameter settings which can be used in the domain.) system. In the next section, we discuss potential ways to make LTR more effective.

**Table 5.** Performance changes in using LTR

Method	NDCG	MAP	P@10
*BM25Opt*	0.457	0.187	0.499
*IROpt*	**0.568**	**0.292**	**0.626**
*IROpt* + LTR	0.539	0.254	0.462

The bold numbers indicate the highest achieved performance.

### Discussion

One of the problems mentioned in the section bioCADDIE challenge overview was the lack of sufficient training data to properly train the LTR models. Small training data at development stage may cause over-fitting (high variance), and at analysis time may cast doubt on the conclusions. To provide the models with more training data, one solution is to use Leave-One-Out cross validation instead of the common k-fold cross validation. To further investigate the changes in performance after applying the steps described in the Methodology: retrieval system architecture and implementation section, we report NDCG, MAP and P@10 for the models with Leave-One-Out cross validation in Tables 6 and 7. Specifically, Table 6 reports the results of using this method to optimize the parameters of the probabilistic information retrieval methods. The highest performance is achieved by the method using ‘*BM25WigExt:* Optimized BM25 + WIG model + external terms (2).’ The changes indicated by α are statistically significant using paired *t*-test (*P* < 0.05).

**Table 6.** Performance results for the steps described in the Methodology: retrieval system architecture and implementation section, with Leave-One-Out cross validation

Model	NDCG	MAP	P@10
*BM25Opt*: Optimized BM25	0.465	0.194	0.495
*BM25Opt-META*: Optimized BM25 without the METADATA field	0.450^α^	0.185^α^	0.481
*BM25Wig:* Optimized BM25 + WIG model	0.563^α^	0.279^α^	**0.629^α^**
*BM25WigBRF:* Optimized BM25 + WIG model + Expansion with BRF (1)	0.559^α^	0.277^α^	0.619^α^
*BM25WigExt:* Optimized BM25 + WIG model + external terms (2)	**0.567**^α^	**0.284**^α^	0.619^α^
*IROpt:* Optimized BM25 + WIG model + Expansion with (1) and (2)	0.561^α^	0.283^α^	0.624^α^

The changes indicated by α, are statistically significant compared to *BM25Opt* using paired *t*-test (p < 0.05).

The bold numbers indicate the highest achieved performance.

**Table 7.** Performance changes in using LTR, with Leave-One-Out cross validation

Method	NDCG	MAP	P@10
*BM25Opt*	0.457	0.187	0.499
*IROpt*	**0.568**	**0.292**	**0.626**
*IROpt* + LTR	0.550	0.272	0.524

The bold numbers indicate the highest achieved performance.

Table 7 reports the performance using the LTR method. When trained on the slightly larger training data offered by the Leave-One-Out cross validation, LTR performance on the NDCG metric improves from 0.539 to 0.550, but still does not reach the performance of the *IROpt* model. The overall improvements are consistent with the results reported with 4-fold cross validation.

Table 8 shows that it is likely that LTR causes ‘overfitting’ if it is used in the same settings. We hypothesize that LTR could be effective if we had access to a noisy judgment about a larger set of queries.

**Table 8.** Retrieval performance for retrained LTR using the extended training data

Method	NDCG	MAP	P@10
*BM25Opt*	0.457	0.187	0.499
IROpt	**0.568**	**0.292**	**0.626**
*IROptLTR: IROpt* + LTR	0.539	0.254	0.462
*IROptLTRExt: IROpt* + LTR	0.552	0.267	0.539

The bold numbers indicate the highest achieved performance.

Noisy labels could be derived from implicit user feedback, such as result clicks, and collected by the search engine, as is commonly done in Web search. In an attempt to examine this hypothesis, we created a very limited and noisy training data as an additional set of 30 queries that were provided, without expert judgments, by the bioCADDIE organizers. We used the *IROpt* model (described in Table 4) to retrieve the top datasets, and tried to label them. A number of criteria were used to consider a dataset to be relevant: The data type and species asked by the query must match the retrieved dataset, otherwise the dataset was labeled irrelevant. Additionally, the presence of the gene and disease names, also indicated moderate relevancy. Finally, biological process descriptions, such as ‘cellular differentiation,’ also can be used as a signal for further investigation. For each query we provided, on average, 5.7 labels in the same scale that was used by the organizers to prepare the official document-query judgements—on average each query in the official training and test sets has 984.1 labels. The judged queries were added to the training parts of the cross validation to retrain LTR framework.

**Figure 3.**
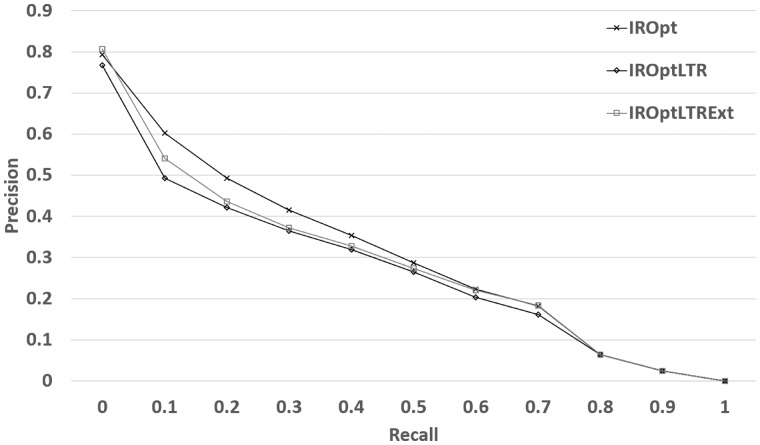
Retrieval performance of the LTR framework when extended training data is used.

Table 8 shows that although LTR still cannot outperform *IROpt* model, with a small effort to increase the training size, we observed a moderate improvement in performance—2.41% in NDCG and 16.66% in terms of P@10 in comparison to our previous LTR model reported in Table 5. The results indicate that implicit user feedback may be helpful—if available in a large scale. Figure 3 shows that the improvement is mostly made in the top retrieved datasets, which is promising specifically for the real world deployment of a search system as most clicked (and viewed) search results are those ranked high by a search system.

In addition to the overall results, we performed cross validation on the extended training set to explore the importance of the different feature groups in the LTR framework (full feature set listed in Table 1). Our goal is to quantify how much each feature group contributes to performance improvements relative to the rest of the features. We performed feature group ablation (by removing one feature group at a time from the model). The results are reported in Table 9. The results show that BM25 scores (feature Group 1), have the highest importance, as they degrade the retrieval results the most when removed. We can also see that Group 6, which consists of the shared bigrams, and Group 4, which consists of the TF-IDF scores for the shared unigrams in query and dataset fields, have the least effect when removed, so can be considered as less important.

**Table 9.** Feature group ablation in learning to rank model

Rank	Category	NDCG after omission
1	(group 1) BM25 scores	0.538
2	(group 3) unigram IDF in the dataset fields	0.544
3	(group 5) unigram in the whole (concatenated) dataset fields	0.548
4	(group 7) DistanceFromStart	0.550
5	(group 2) unigram TF in the dataset fields	0.550
6	(group 8) DomainWeight	0.553
7	(group 6) shared bigrams	0.557
8	(group 4) unigram TF-IDF in the dataset fields	0.558

Groups are mentioned in Table 1.

To measure the effectiveness of the external resources used in the query expansion step, we re-trained *BM25WigExt* model (described in Table 4) once using web search (Wikipedia and NCBI) and once using the API (HGNC and KEGG). Table 10 reports the improvement, which is achieved in each case. The experiment shows that expansion using the API is mainly improving P@10. On the other hand, expansion using web search improves the overall retrieval performance (NDCG and MAP).

**Table 10.** Performance improvements using external expansion resources

External expansion resource	NDCG	MAP	P@10
API	0.534	0.259	**0.612**
Web search	0.547	**0.272**	0.586
Web search + API	**0.547**	**0.272**	0.590

To gain intuition and insights into our system performance, we report case studies of applying the different system variations on the retrieval performance, in Table 11. Specifically, we report the results for the specific queries, identified as 1, 3, 10 and 15 in the official test set, and the performance changes before and after each modification (using the *IROpt* model as the underlying first-stage retrieval method). Query terms which are marked by ‘**+**’ are keywords which are detected by the *WIG* model and assigned the highest weight in the query. Query terms (pre-processed to remove inflections, or stemmed) enclosed in ‘**[]**’ were added by *external expansion*, and query terms (stemmed) enclosed in ‘**<>**’were added by *BRF*.

**Table 11.** Changes in retrieval performance before and after query modification for query numbers 1, 3, 10, and 15. Query terms enclosed in ‘**[]**’ are added using external resources, and query terms enclosed in ‘**<>**’are added by BRF. Query terms marked by ‘**+**’ are keywords with the highest weight in WIG model

Query No	Original query terms and automatically expanded terms	NDCG before modification	NDCG after modification
1	Find protein sequencing data related to bacterial^+^ chemotaxis^+^ across all databases^+^**[**citat cell bacteria gradient direct respons develop system primari organ**] <**nifh ncbi thaw permafrost alaskan 5s harbor 23 bigsdb campylobact**>**	0.111	0.291 (+162%)
3	Search for all data types related to gene TP53INP1^+^ in relation to p53^+^ activation across all databases^+^**[**cell protein express cancer tumor induc function apoptosi human dna**] <**ptm mmtv ncbi ra sequenc muscl ebi salivari restrict express**>**	0.342	0.710 (+107%)
10	Search for data of all types related to energy metabolism^+^ in obese^+^ M. musculus^+^**[**fat studi gene profil cell**] <**fat obstrut massag apneic simpl n apnea sleep mechan therapy**>**	0.373	0.436 (+16%)
15	Find data on the NF-kB^+^ signaling pathway in MG (Myasthenia^+^ gravis^+^) patients **[**activ cell 2 rna gene**] <**nfkbiz stat3 thymoma dlbcl protein myc ncbi abc oci sequenc**>**	0.603	0.524 (-13%)

We can see that in most cases the automatically added terms are relevant to the original query terms. For example, in query number 3, the new terms ‘protein,’ ‘cancer,’ ‘tumor’ and ‘human’ are added to the query which is directly related to gene TP53INP1. We can also observe that *WIG* model succeed in detecting important query terms most of the times. Detecting the keywords (by assigning a higher weight to them), and discarding the less informative words are the main reasons for making 17.06% improvement on the NDCG metric, reported in Table 4.

In summary, we have explored a variety of information retrieval methods, ranging from probabilistic information retrieval models such as automated query term weighting (*WIG*) and BRF for query expansion, as well as the state-of-the-art learning to rank (LTR) machine learned algorithms for automatically learning the ranking functions. We showed that the use of automatically collected meta-data (e.g. website description of the source database from which the datasets were derived) was helpful for providing additional general information about dataset topic. Similarly, it may be possible to incorporate meta-data about the probability of a match between the genes mentioned in the query by name, and the actual data content of the dataset, as proposed in reference (18). This match could be incorporated as a feature, together with the textual matches, into the general ranking models described in this paper.

Interestingly, our results show that with automatic optimization and parameter tuning, traditional probabilistic information retrieval methods outperform the machine-learned approaches. This is due, in this case, to the small amounts of labeled training data available through the bioCADDIE Challenge. However, we showed that adding even a moderate amount of additional training data improves the machine-learned ranking (LTR), which suggests a promising area of future work to incorporate additional noisy training data in the form of clicks and other feedback from the users of the system, e.g. as in Ref. (19).

## Conclusions

We described the system implementation and experiments used to evaluate the performance of an information retrieval system for finding relevant biomedical datasets, as part of the bioCADDIE Dataset Retrieval Challenge. Specifically, we experimented with optimizing traditional probabilistic information retrieval techniques such as fielded BM25 metrics, query term boosting and automatic (blind) relevance feedback, as well as with the machine learning-based ‘learning to rank’ approach for automatically discovering the ranking functions from a rich representation of the query and datasets. Our results showed significant improvements that can be achieved with careful optimization of the traditional information retrieval methods, while demonstrating that, at least for the bioCADDIE challenge, machine learning-based techniques do not yet outperform the traditional information retrieval techniques. Extensive analysis was performed to understand the benefits of the different system variations, providing examples and case studies of specific improvements. Together, the methods, results and the analysis presented in this paper, as well as the provided data and code (The code and data is available at: https://github.com/emory-irlab/biocaddie) released to the research community, provide a valuable resource for biomedical researchers, practitioners and developers who could build on our work for similar biomedical dataset retrieval tasks.

Going forward, we believe that with additional training data, and by using additional sources of evidence such as the actual dataset contents and user feedback as features, we could further improve the retrieval performance for this challenging task.

## Funding

This work was partially funded by NIH Grant 1U24AI117966-01 (Year 3 Pilot projects), and by Emory University.

*Conflict of interest*. None declared.
